# Guest Editorial. Interdisciplinary Approaches for Human Cognition: Expand ing Perspectives on the Mind

**DOI:** 10.21500/20112084.7351

**Published:** 2024-07-01

**Authors:** Jaime A. Riascos Salas, Vinícius Rosa Cota, Hernán Villota, Daniel Betancur Vasquez

**Affiliations:** 1 Institución Universitaria de Envigado (IUE), Envigado, Colombia. Institución Universitaria de Envigado Institución Universitaria de Envigado (IUE) Envigado Colombia; 2 Rehab Technologies Lab, Istituto Italiano di Tecnologia, Genoa, Italy. Rehab Technologies Lab Istituto Italiano di Tecnologia Genoa Italy

The brain is one of the most intriguing and complex systems we know so far. The quest to decipher its intricate workings continues to captivate scientists and researchers worldwide. By comprehending the brain’s capacities, functions, connectivity, origins, and development, we open doors to precise health treatments and technological advancements that enhance our well-being. One crucial pillar in this endeavor is fostering interdisciplinary discussions. We can create a holistic and comprehensive understanding of the brain by bringing together professionals and researchers from diverse fields. Collaboration and knowledge sharing become the keys to unraveling the enigmas of the brain.

In this endeavor, the Latin American Workshop on Computational Neuroscience (LAWCN) plays a pivotal role by fostering a vibrant community committed to understanding the intersection of neuroscience, computa tional approaches, and cutting-edge technological innovations. LAWCN was born in 2012 at the Federal University of Rio Grande do Sul (UFRGS) where it later formally became a biannual event in 2017. Since its beginning, its objective has been to support collaborative learning and knowledge sharing through events hosted in unique Brazilian cities such as Porto Alegre (2017), São João del-Rei (2019), and São Luís de Maranhão (2021).

The 4th Latin American Workshop on Computational Neuroscience (IV LAWCN, https://lawcn.co/) was held from November 28^th^ to 30^th^, 2023, in Envigado, Antioquia, Colombia. This edition represents a significant milestone, as it was the first time outside its Brazilian origins. The success of the IV LAWCN is attributed to the collaboration between the University of Envigado (IUE), led by the Faculty of Engineering and its research group “Grupo de Investigación Tecnologías Emergentes Sostenibles e Inteligentes- GITESI”, and the Colegio Colombiano de Neurociencias (COLNE). This collaboration reflects their dedication to advancing computational neuroscience research in Colombia. Furthermore, the continued support from the International Brain Research Organization (IBRO) was fundamental, providing funding for travel grants, promoting accessibility for international researchers, and facilitating a roundtable to discuss current and future challenges of gender diversity in neuroscience.

The IV LAWCN provided an environment for knowledge exchange, network building, and research collaboration, with 250 participants attending the oral presentation of up to 28 research papers and 15 posters. The conference featured nine distinguished keynote speakers from multiple countries such as France, the USA, Norway, Brazil, and Colombia, offering a diverse international perspective. Additionally, LAWCN had an insightful presentation from the Federation of Latin American and Caribbean Neuroscience Associations (FALAN), a roundtable on gender diversity, the launch of a Brain- Computer Interface LATAM community, and a short course in computational modeling using NetPyNE.

This volume of the International Journal of Psychological Research contains a selection of exceptional research presented at the IV LAWCN, rigorously reviewed by a minimum of three experts from our Program Com mittee (PC). The volume includes papers on the following topics:

## Neurophysiology and Neuroscience

This section delves into the fundamental mechanisms that shape the brain’s intricate operations. Investigations explore the neural basis of sensory perception, particularly the visual system, using both accessible electrophysiological techniques (Isaza et al., 2024) and advanced theoretical modeling (Vasconcelos et al., 2024). These studies aim to decode how the brain processes and interprets visual information, laying the groundwork for advancements in diagnostics and potential interventions. Also, new perspectives are raised and discussed about the fundamental behavior of the human brain (Gomez Molina, 2024).

## Quantitative Psychology

The multifaceted nature of the human mind is the core focus of this section. Research papers in this area examine the intricate relationship between cognitive processes like attention and motivational factors such as self-efficacy (Cardoso et al., 2024). Additional studies employ novel combinations of electrophysiological measures and psychological inventories to assess learning processes, potentially leading to refined educational strategies and a deeper understanding of how to optimize human potential (Rios, 2024).

## Al Applications in Neuroscience and Neurology

The transformative impact of artificial intelligence (AI) in healthcare is underscored in this section. Researchers leverage the power of deep learning architectures to propose the diagnosis and treatment of neurological disorders. These sophisticated computational models aid in the detection and analysis of conditions like stroke (Martinez et al., 2024a) and Parkinson’s disease (Martinez et al., 2024b), driving earlier and more tailored interventions. Additionally, Al-powered systems pave the way for early detection of addictive behaviors (Soler et al., 2024) and demonstrate great promise in enhancing emotional regulation through the integration of neurofeedback, virtual reality, and machine learning (Rodriguez et al., 2024).

## Neural Signal Processing

This section showcases sophisticated analytical tools and methodologies tailored for the interpretation of the brain’s complex electrical activity. The development of open- source software frameworks facilitates vital research into neurological conditions like epilepsy, potentially accelerating the discovery of new treatments (Rodrigues et al., 2024). Moreover, studies delving into the neural underpinnings of social cognition investigate implicit biases, employing advanced signal analysis techniques to offer novel approaches for understanding and potentially addressing social prejudice (Quiza-Montealegre et al., 2024).

The contributions within this volume offer a compelling glimpse into the rapidly advancing field of computational neuroscience. The innovative research presented at IV LAWCN demonstrates the growing synergy between experimental neuroscience, data science, and emerging technologies. We anticipate that these cross-disciplinary collaborations will continue to yield breakthroughs, resulting in improved diagnostic tools, more effective treatments, and a profound understanding of the human brain’s extraordinary capabilities. Finally, we express our deepest gratitude to our esteemed strategic partners (Universidad de Antioquia, Universidad Escuela de Ingenierías de Antioquia, Universidad Remington, Universidad de San Buenaventura Medellin, Universidad CES, Universidad Cooperativa de Colombia, University of Florida, Université de Tours, Université dOrléans and Inserm), keynote speakers, authors, reviewers, and all participants that make this remark able conference possible.

